# Aldose reductase interacts with AKT1 to augment hepatic AKT/mTOR signaling and promote hepatocarcinogenesis

**DOI:** 10.18632/oncotarget.17791

**Published:** 2017-05-10

**Authors:** Jia-Xing Zhao, Ya-Wei Yuan, Cheng-Fu Cai, Dong-Yan Shen, Mao-Li Chen, Feng Ye, Yan-Jun Mi, Qi-Cong Luo, Wang-Yu Cai, Wei Zhang, Ying Long, Yong Zeng, Guo-Dong Ye, Shu-Yu Yang

**Affiliations:** ^1^ State Key Laboratory of Cellular Stress Biology, Innovation Center for Cell Biology, School of Life Sciences, Xiamen University, Xiamen, Fujian, 361002, China; ^2^ State Key Laboratory of Marine Environmental Science, College of Ocean and Earth Sciences, Xiamen University, Xiamen, Fujian, 361002, China; ^3^ The First Affiliated Hospital, Medical College, Xiamen University, Xiamen, Fujian, 361003, China; ^4^ School of Pharmaceutical Science, Xiamen University, Xiamen, Fujian, 361003, China; ^5^ Medical College, Xiamen University, Xiamen, Fujian, 361003, China; ^6^ Translational Medicine Center, Hunan Cancer Hospital, Changsha, Hunan, 410013, China

**Keywords:** aldose reductase, AKT/mTOR signaling, hepatocellular carcinoma

## Abstract

Marked up-regulation of aldose reductase (AR) is reportedly associated with the development of hepatocellular carcinoma (HCC). We investigated how aberrantly overexpressed AR might promote oncogenic transformation in liver cells and tissues. We found that overexpressed AR interacted with the kinase domain of AKT1 to increase AKT/mTOR signaling. In both cultured liver cancer cells and liver tissues in DEN-induced transgenic HCC model mice, we observed that AR overexpression-induced AKT/mTOR signaling tended to enhance lactate formation and hepatic inflammation to enhance hepatocarcinogenesis. Conversely, AR knockdown suppressed lactate formation and inflammation. Using cultured liver cancer cells, we also demonstrated that AKT1 was essential for AR-induced dysregulation of AKT/mTOR signaling, metabolic reprogramming, antioxidant defense, and inflammatory responses. These findings suggest that aberrantly overexpressed/over-activated hepatic AR promotes HCC development at least in part by interacting with oncogenic AKT1 to augment AKT/mTOR signaling. Inhibition of AR and/or AKT1 might serve as an effective strategy for the prevention and therapy of liver cancer.

## INTRODUCTION

Aldose reductase (EC1.1.1.21, AKR1B1, AR) is a member of the aldo-keto reductase (AKR) protein family, which plays important roles in nuclear receptor signaling, inflammatory responses, osmoregulation, endobiotic and xenobiotic detoxification, hormone synthesis and action, cellular metabolism and reproduction *etc*. [1]. For glucose metabolism, AR serves as the first and the rate-limiting enzyme for the polyol pathway (PP) to reduce glucose to sorbitol, while sorbitol is further oxidized by sorbitol dehydrogenase (SDH) to generate fructose [2].

In the liver, *AR* was found to be transiently expressed during embryogenesis [3]. In adult animals, hepatic *AR* expression or activity is barely detectable or absent [3, 4]. A number of recent studies, nonetheless, have shown that hepatic AR can be significantly induced and activated under a variety of stress conditions or in diseased livers. In humans or rodents, *AR* and aldo-keto reductase family 1B10 (*AKR1B10*, also known as AR-like-1), were often among the most significantly up-regulated genes in many types of cancers including cervical cancer, colon cancer, leukemia, pancreatic cancer and hepatocellular carcinoma (HCC) [4–11]. Consistent with the transcriptomic analyses, many proteomic studies also indicated very significant elevations in the protein expression of AR and AKR1B10 in human liver cancer tissues [5, 7, 8, 12]. More interestingly, some reports showed that AR/AKR1B10 mRNA expression levels is an independent predictor of prognosis in HCC patients [10, 11]. In spite of these studies, however, very little studies were focus on the mechanism of the aberrantly overexpressed *AR/AKR1B10* contribute to the development or progression of various types of cancers.

Potential mechanisms are aberrant overexpression/activation of hepatic AR and/or Polyol Pathway (PP)-associated overt oxidative stress and inflammation, which are believed to contribute significantly to the development of cancers [4, 13, 14]. Studies also suggest that inhibition of oxidative stress or inflammation is helpful with cancer prevention or treatment. For instance, trans-aldolase deficiency-induced hepatocarcinogenesis was associated with activation of AR that can be prevented by treatment with N-acetylcysteine [15]. In rats, diethylnitrosamine (DEN)-induced hepatocarcinogenesis was also associated with activation of AR and treatment with a ROS scavenger dially sulfide significantly ameliorated DEN-induced HCC [16].

Aberrant overexpression/activation of hepatic AR/PP may also contribute to lactate over-production, as in the well-known “Warburg effect” or aerobic glycolysis, whereby cancer cells exhibit increased conversion of glucose to lactate, even in the presence of sufficient oxygen [17]. Aberrant AR/PP-mediated hepatic over-production of fructose were shown to reprogram cellular glucose-lipid metabolism to significantly affect the development of obesity, metabolic syndrome, nonalcoholic fatty liver disease, and nonalcoholic steatohepatitis [18–21], all of which are important risk factors for the development of HCC. Fructose by itself was suggested to be able to promote tumorigenesis, in part by inducing metabolic reprogramming and lactate over-production [22–25]. However, the relationship between AR and lactate-production/Warburg effect has been unclear.

In the present study, we investigated the potential roles of AR in the development of HCC. The effects of AR overexpression and AR knockdown/knockout on lactate formation, the expression of inflammatory cytokines, and the most important Warburg effect regulating pathway, the AKT/mTOR signaling pathway, were evaluated *in vitro* in cultured liver cancer cells and *in vivo* in the livers of DEN-induced transgenic HCC model mice.

## RESULTS

### Overexpression of AR enhanced whereas knockdown of AR suppressed cancer cell proliferation, colony formation, and migration, invasion and wound-healing

**Figure 1:**
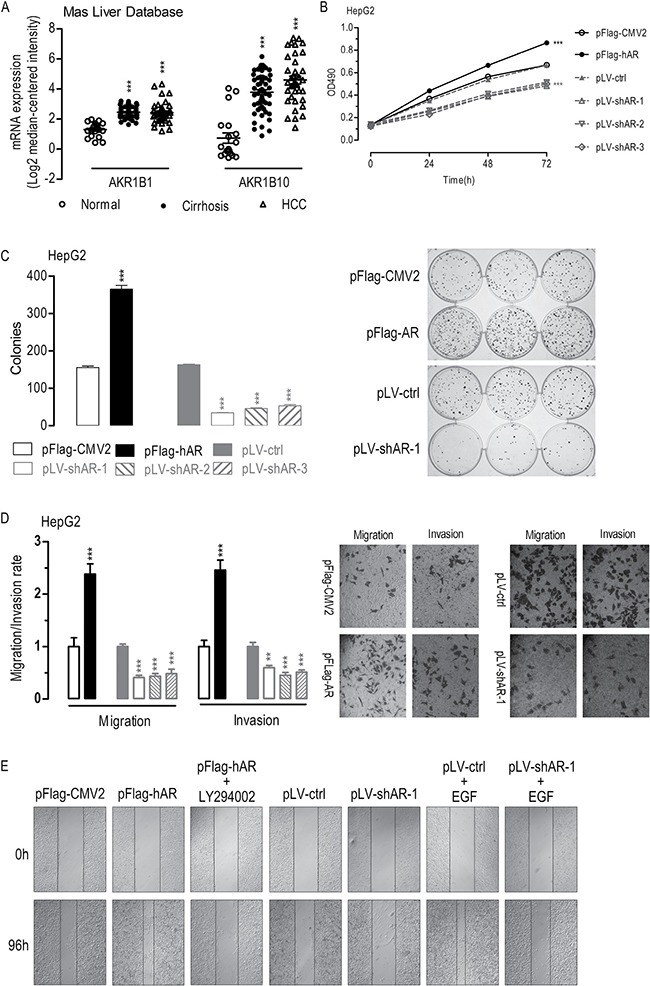
Effects of AR overexpression or knockdown on cell proliferation, migration, invasion, colony formation, and wound-healing in HepG2 cells Dot plots showing *AR* and *AKR1B10* mRNA expression in clinical liver samples as studied by Mas *et al*. (**A**) Overexpression of *AR* enhanced whereas knockdown of *AR* suppressed cell proliferation (**B**) (*n* = 6), colony formation (**C**) (*n* = 6), migration and invasion (**D**) (*n* = 6), and wound healing (**E**) (*n* = 3). Data were expressed as the mean ± SEM. **p < 0.01; ***p < 0.001, compared to pFlag-CMV2 or pLV-ctrl transfected cells.

In humans, microarray analyses identified *AR* and *AKR1B10* mRNA up-regulated in the development of hepatitis C virus (HCV)-associated HCC [26, 27]. *AR* mRNA ranked at the top 3% and 7% of the significantly altered genes in HCV-positive HCC and HCV-positive cirrhosis respectively, in comparison with the HCV-negative normal subjects (Figure 1A, *p* < 0.001). Meanwhile, *AKR1B10* mRNA ranked at the top 2% and 9% of the significantly altered genes in HCC and cirrhosis respectively (*p* < 0.001).

To evaluate the effects of *AR* overexpression or knockdown on hepatocarcinogenesis, we performed transfection studies using HepG2 or SMMC-7721 liver cancer cells with a plasmid overexpressing *AR* or three plasmids overexpressing shRNAs against *AR* (Supplementary Table 1). In HepG2 cells, *AR* overexpression significantly enhanced HepG2 cell proliferation (Figure 1B), colony formation (Figure 1C), migration and invasion (Figure 1D), and wound-healing (Figure 1E). In contrast, shRNA-mediated knockdown of *AR* significantly suppressed HepG2 cell proliferation (Figure 1B), colony formation (Figure 1C), migration and invasion (Figure 1D), and wound-healing (Figure 1E). Remarkably, inhibition the phosphorylation of AKT1 by LY294002 significantly suppressed *AR*-induced wound-healing (Figure 1E) [28]. Furthermore, although small but not significant difference in wound-healing was found between the *AR* knockdown cells (pLV-shAR-1 transfected) and the control cells (pLV-ctrl-transfected), knockdown of *AR* suppressed wound-healing in EGF-stimulated cells (pLV-ctrl+EGF versus pLV-shAR-1+EGF) [29]. Similar effects of *AR* overexpression or knockdown on cell proliferation, colony formation, migration and invasion, and wound-healing observed in SMMC-7721 liver cancer cells (Supplementary Figure 1). Together these results suggested that aberrantly overexpressed *AR* promote oncogenic transformation or metastasis.

### Overexpression of AR stabilized whereas knockdown of AR destabilized protein expression of AKT1 and AKT2

**Figure 2:**
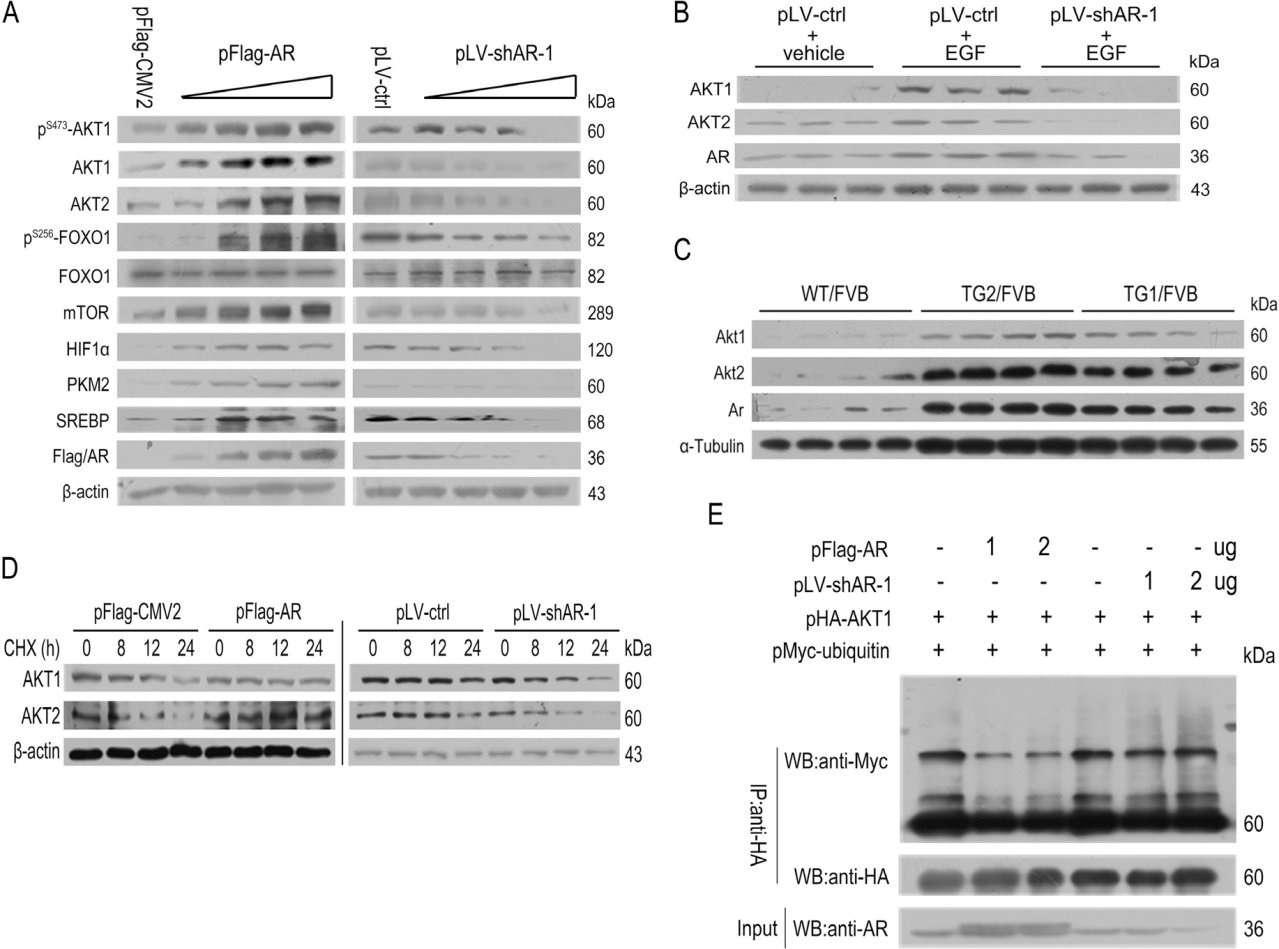
The effects of AR overexpression or knockdown on AKT/mTOR signaling (**A**) Overexpression of *AR* enhanced whereas knockdown of *AR* suppressed the proteins expression of AKT/mTOR pathway. (**B**) Knockdown of *AR* prevented EGF-stimulated up-regulation of AKT1 and AKT2 (*n* = 3). (**C**) Stabilization of Akt1/Akt2 by *AR* overexpression in 34 wk old male TG1/FVB and TG2/FVB mice (*n* = 4). (**D**) Overexpression of *AR* stabilized AKT1/2 whereas knockdown of *AR* destabilized AKT1/2. (**E**) Overexpression of *AR* suppressed AKT1 ubiquitination whereas knockdown of *AR* promoted AKT1 ubiquitination.

Since *AR*-induced wound-healing was largely suppressed by LY294002 treatment, we investigated how *AR* might affect the activity or expression of AKT1 and AKT2. Our qPCR analyses revealed that both *AR* overexpression and *AR* knockdown in HepG2 cells did not affect the mRNA expression of *AKT1* and *AKT2* (Supplementary Figure 2). Western blot analyses indicated that serine-473 phosphorylated AKT1 (p^S473^-AKT1), AKT1, AKT2, and several AKT1 and AKT2 down-stream signaling proteins, including serine-256 phosphoryalted-FOXO1 (p^S256^-FOXO1) and the key pathway of Warburg Effects [30] (including mTOR [31], HIF-1α [32], PKM2 [32], and another mTOR-regulated protein SREBP [33]), dose-dependently up-regulated in HepG2 cells overexpressing *AR*, with the exception of FOXO1 (Figure 2A). Consistent with this, these proteins dose-dependently down-regulated in *AR*-knockdown cells.

Consistent with previous publication [29, 34, 35], knockdown of *AR* suppressed EGF-induced up-regulation of AKT1 and AKT2 in HepG2 cells (Figure 2B). *In vivo*, the Akt1 and Akt2 proteins significantly increased in liver tissues of liver-specific human *AR*-overexpressing transgenic FVB mice (TG1/FVB and TG2/FVB) (Figure 2C).

In the case of Akt1/2/3 isoforms have the similar structure (Supplementary Figure 3), following experiments performed using human Akt1 as a representation of Akt family. Also previous studies of mice showed that Akt1 were more important to cell growth, whereas Akt2 mediated glucose metabolism [36–38], this study focused on Akt1 only. Further co-immunoprecipitation analyses indicated that *AR* overexpression markedly suppressed the binding of MYC-ubiquitin to AKT1, whereas this effect was not significantly in *AR* knockdown cells (Figure 2D and 2E). *AR* overexpression thus might stabilized AKT1 in part through preventing proteosome-mediated degradation of ubiquitinated AKT1.

### AR interacted with the kinase domain of AKT1

**Figure 3:**
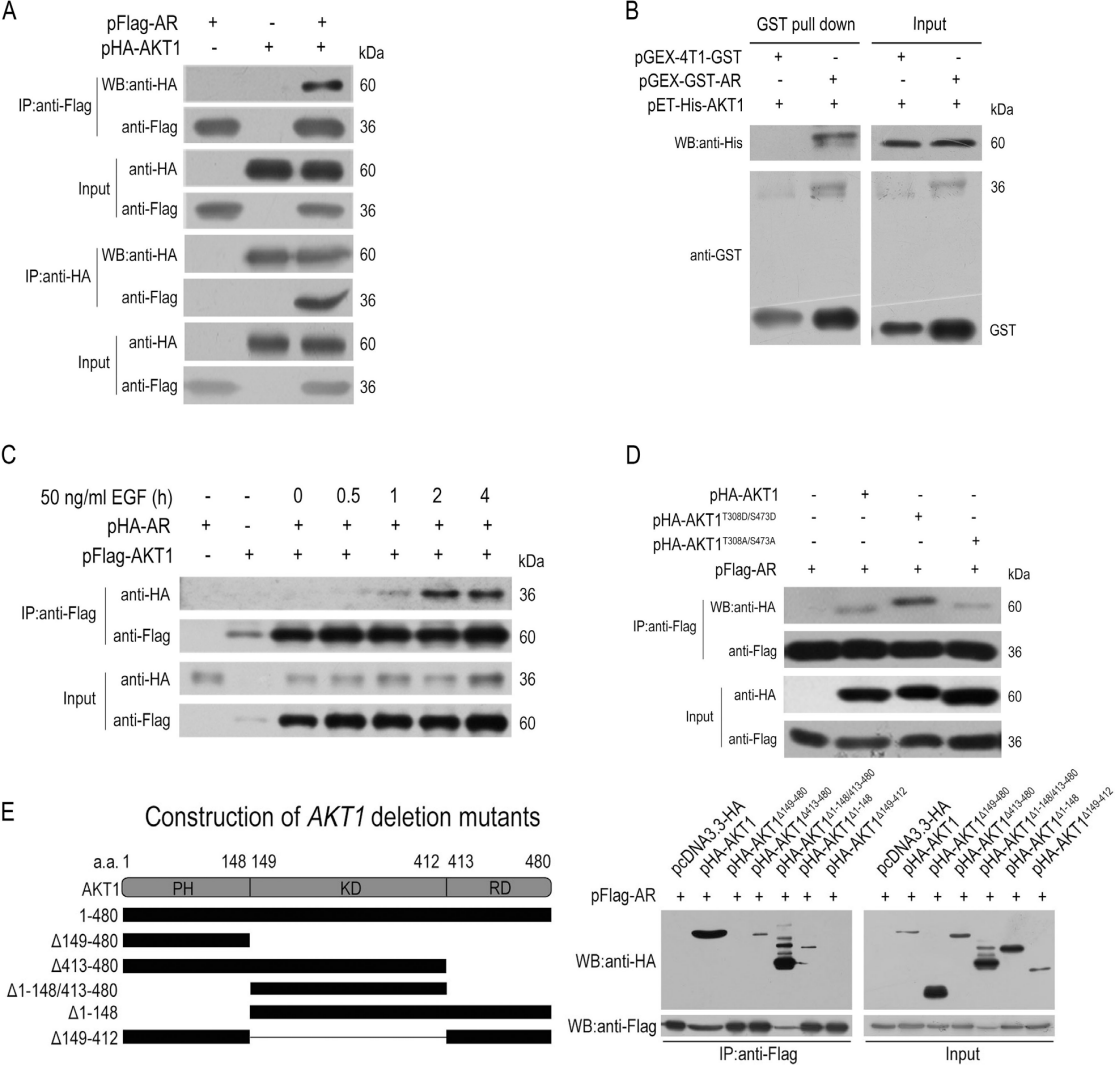
AKT1 interacts with AR physically (**A**) Co-immunoprecipitation between the plasmid-encoded AKT1 and AR in HEK293T cells using anti-Flag or anti-HA antibodies. (**B**) *In vitro* GST pull-down of AR-AKT1 (both expressed in bacteria) complex. (**C**) EGF treatment time dependently enhanced the AR-AKT1 interaction. (**D**) Constitutively-active AKT1 (HA-AKT1^T308D/S473D^) had a higher affinity for AR than the WT AKT1 or constitutively inactive AKT1 (HA-AKT1^T308A/S473A^). (**E**) AR physically interacts with the kinase domain (KD) but not the PH/helix or the RD domain of AKT1.

To further explore the molecular mechanisms of AR stabilized AKT1 and AKT2, immunoprecipitation (IP) assays were performed. In HEK293T cells, Flag-tagged AR co-precipitated with HA-tagged AKT1, using either anti-Flag or anti-HA antibody (Figure 3A). Furthermore, the *E. coli* expressing His-tagged AKT1 combined *E. coli* expressing GST-tagged AR but not the empty vector pGEX-4T1-GST (Figure 3B).

Also by co-immunoprecipitation analyses, the AR-AKT1 interaction enhanced time-dependently by EGF treatment in HepG2 cells (Figure 3C). Moreover, AR had a higher affinity for a constitutively-active AKT1 (AKT1^T308D/S473D^) than either the WT AKT1 or a constitutively-inactive AKT1 (AKT1^T308A/S473A^) (Figure 3D).

Then, co-immunoprecipitation analyses were tested using 3 truncated mutants of *AR* and 5 truncated mutants of *AKT1* (Supplementary Table 2) [39]. Probably due to the lack of distinctive structural domains [40, 41], three AR deletion mutants co-precipitated with the wildtype (WT) AKT1 protein, although the N-terminal deletion mutant (Flag-AR^Δ1–100^) had a much weaker affinity (Supplementary Figure 4). In contrast to this, HA-AKT1^D413–480^, HA-AKT1^Δ1–148/413–480^ and HA-AKT1^Δ1–148^ co-precipitated with the WT AR protein, but not HA-AKT1^Δ149–480^ and HA-AKT1^Δ149–412^ (Figure 3E), which indicated that the kinase domain of AKT1 alone was sufficient for the direct protein-protein interaction with AR.

### AKT1 was essential for AR-induced significant alterations in AKT/mTOR signaling, lactate formation, and TNFα/IL-6 mRNA expression

**Figure 4:**
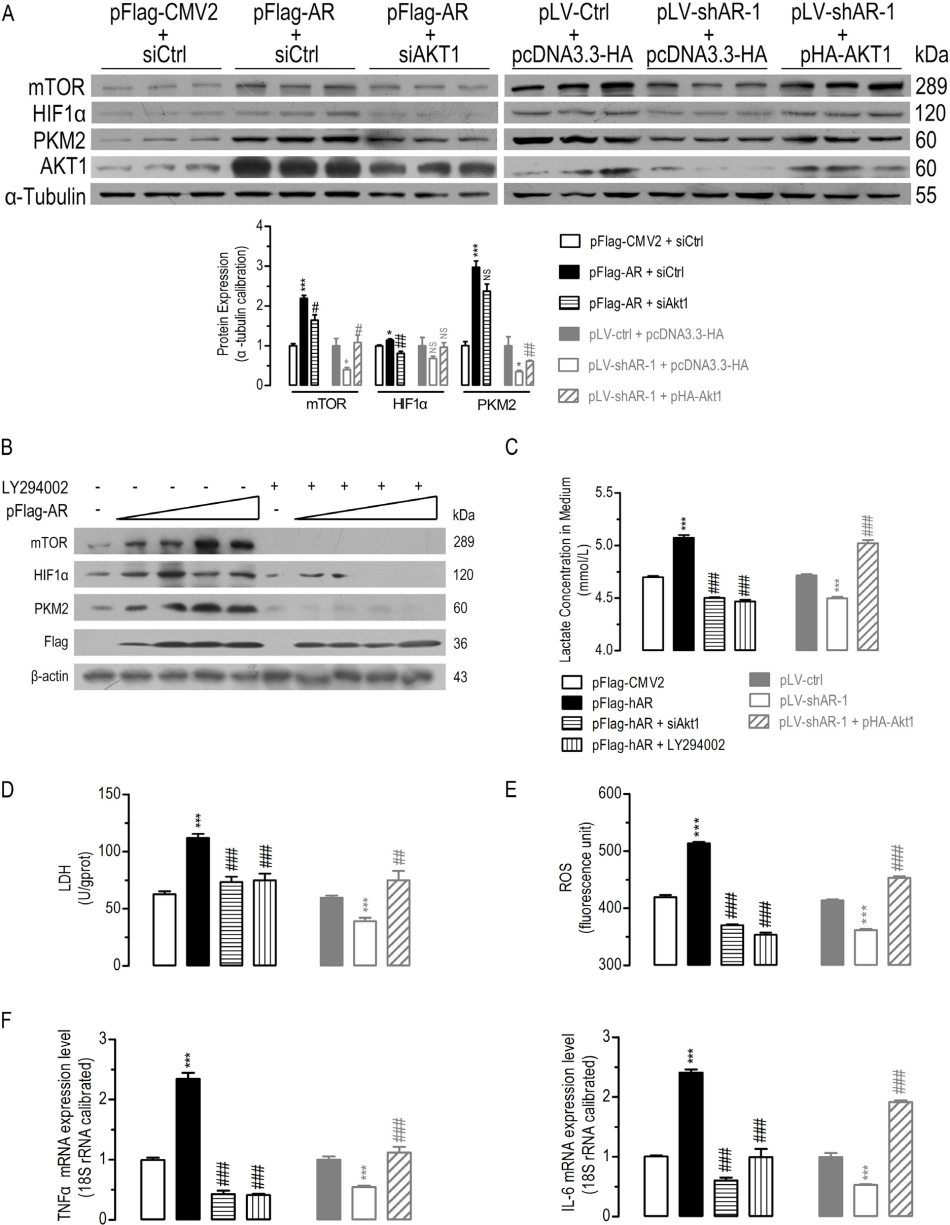
AKT1 was essential for AR-induced alterations in AKT/mTOR signaling, lactate formation and TNFα/IL-6 mRNA expression AKT1 was essential for AR-induced significantly disturbed protein expression of mTOR, HIF1a, and PKM2 in HepG2 cells (**A**) (*n* = 3). Inhibition of AKT1 phosphorylation by LY294002 significantly diminished AR overexpression-induced mTOR, HIF1α, and PKM2 protein expression (**B**). AKT1 was essential for AR-induced significantly disturbed lactate formation (**C**) (*n* = 6), LDH activity (**D**) (*n* = 6), ROS (**E**) (*n* = 6), *TNF*a*/IL-6* mRNA expression (**F**) (*n* = 6) in HepG2 cells. Data were expressed as the mean ± SEM. NS, not significant; *p < 0.05; **p < 0.01; ***p < 0.001 (compared to pFlag-CMV2+siCtrl or pLVctrl+pcDNA3.3-HA transfected cells); ^#^*p* < 0.05; ^##^*p* < 0.01; ^###^*p* < 0.001 (compared to pFlag-AR+siCtrl or pLV-shAR-1+pcDNA3.3-HA transfected cells).

To evaluate the effects of AKT1 in AR-induced hepatocarcinogenesis, transfection rescue studies were performed in HepG2 cells. In HepG2 cells, *AR* overexpression-induced AKT1, mTOR, HIF1a and PKM2 protein expression up-regulation (pFlag-CMV2+siCtrl transfected versus pFlag-AR+siCtrl transfected) was significantly diminished in cells co-treated with siAKT1 (pFlag-AR+siCtrl transfected versus pFlag-AR+siAKT1 co-transfected) (Figure 4A). Conversely, *AR* knockdown-induced AKT1, mTOR, and PKM2 down-regulation (pLV-ctrl+pcDNA3.3-HA transfected versus pLV-shAR-1+pcDNA3.3-HA transfected) was significantly restored in AKT1 co-overexpression cells (pLV-shAR-1+pcDNA3.3-HA transfected versus pLV-shAR-1+pHA-AKT1 co-transfected), although HIF1a were not altered significantly. Additionally, inactivation of AKT1 by LY294002 also reversed AR-induced mTOR, HIF1a and PKM2 up-regulation (Figure 4B).

In comparison with the control cells, *AR* overexpression increased lactate formation (Figure 4C) and LDH activity (Figure 4D), *AKT1* knockdown by siRNA (siAKT1) or inactivation by LY294002 treatment significantly diminished *AR* overexpression-induced lactate formation and LDH activity increasing. Meanwhile, *AR* knockdown suppressed lactate formation and LDH activity, but *AKT1* overexpression significantly restored *AR* knockdown-induced lactate formation and LDH activity decreasing.

Since AR and AKT both regulated ROS and inflammatory signals [42–46], TNFα and IL-6 mRNA were analyzed by qPCR. Knockdown/inactivation AKT1 inhibited AR overexpression-induced ROS (Figure 4E) and *TNFα/IL-6* mRNA expression increasing (Figure 4F). Whereas overexpression of *AKT1* restored *AR* knockdown-induced ROS and *TNFα/IL-6* mRNA expression decreasing. These *in vitro* experiments suggested that AKT1 was essential for AR-induced dys-regulations in AKT/mTOR signaling, metabolic reprogramming, antioxidant defense and inflammatory responses in HCC cells.

### Liver-specific AR overexpression tended to promote whereas Ar deficiency tended to suppress DEN-induced HCC

**Figure 5:**
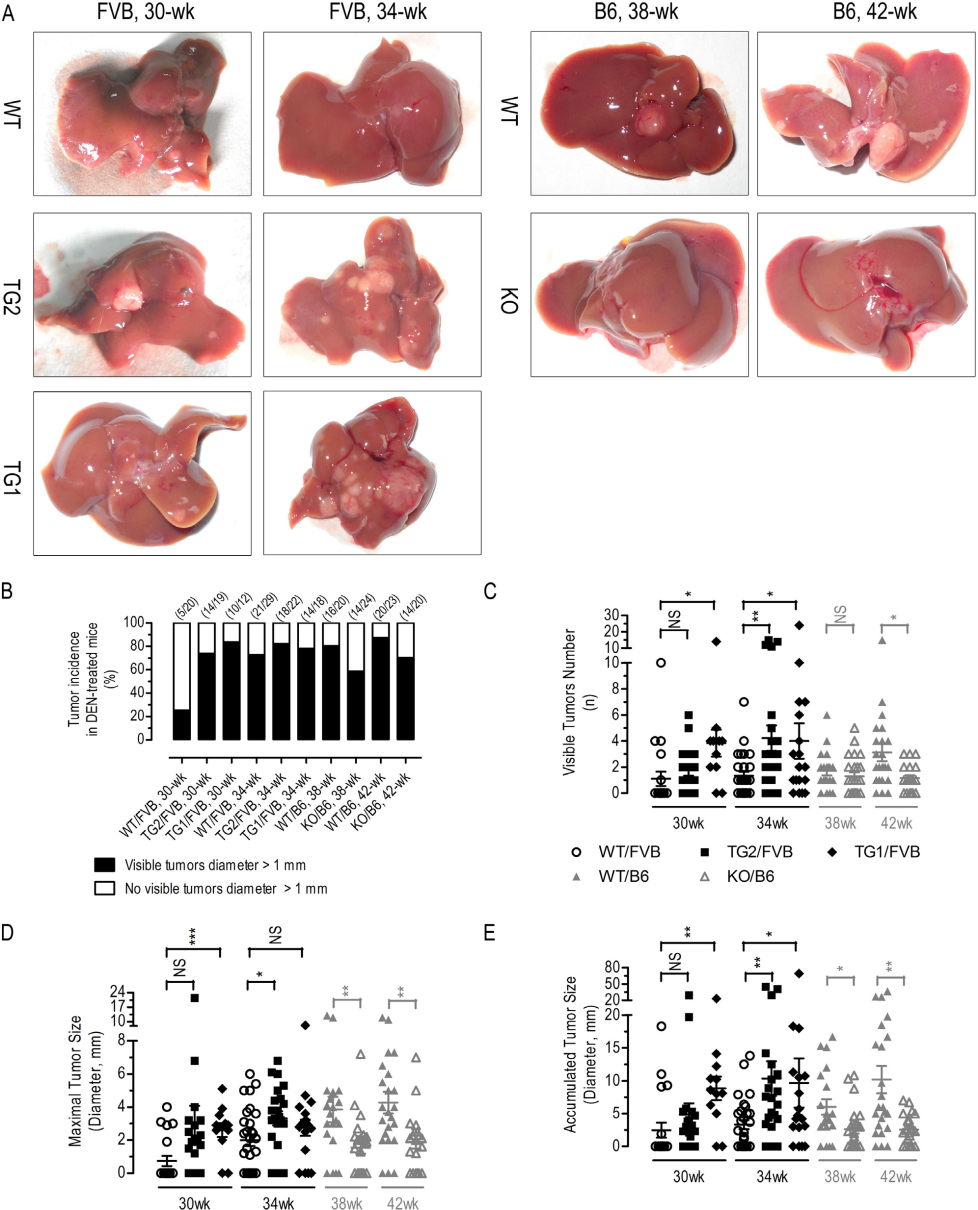
DEN-induced HCC in liver-specific AR overexpressing transgenics and Ar knockout mice Typical liver photos (**A**) tumor incidence (**B**) (*n* = 12–29), visible tumor number (**C**) (*n* = 12–29), maximal tumor size (**D**) (*n* = 12–29), and accumulated tumor size (**E**) (*n* = 12–29) of different groups of DEN-treated mice. Numerical data were expressed as the mean ± SEM. NS, not significant; *p < 0.05; **p < 0.01; ***p < 0.001 (TG2/FVB or TG1/FVB versus WT/FVB, KO/B6 versus WT/B6).

To examine the effects of *AR* regulated hepatic Akt/mTor signaling, lactate formation, inflammatory response gene expression and liver cancer development *in vivo*, HCC was induced in 2-wk old WT/FVB, TG2/FVB, TG1/FVB, WT/B6 and KO/B6 [47, 48] male mice by a single injection of DEN at the dosage of 25 mg/kg body weight. In FVB mice, DEN-treated *AR*-overexpressing mice (TG2/FVB, TG1/FVB) had significantly higher tumor incidence (% mice with tumors > 1 μm), visible tumor number per mouse, maximal tumor size and accumulated tumor size (in diameter, mm) than the DEN-treated control mice (WT/FVB) (Figure 5), but not in body or liver weight (Supplementary Figure 5). Conversely, a significant amelioration in tumor incidence, visible tumor number per mouse, maximal tumor size and accumulated tumor size was observed in *Ar* deficient B6 mice (KO/B6) as compared to the control mice (WT/B6) (Figure 5), with a few minor exceptions. Together, these data suggested that liver-specific *AR* overexpression promote whereas *Ar* deficiency/knockdown suppress HCC tumorigenesis or progression.

### Significant alterations in Akt/mTor signaling, lactate formation, and Tnfα/Il-6 mRNA expression in the liver tissues of AR-overexpressing transgenic and Ar knockout mice

**Figure 6:**
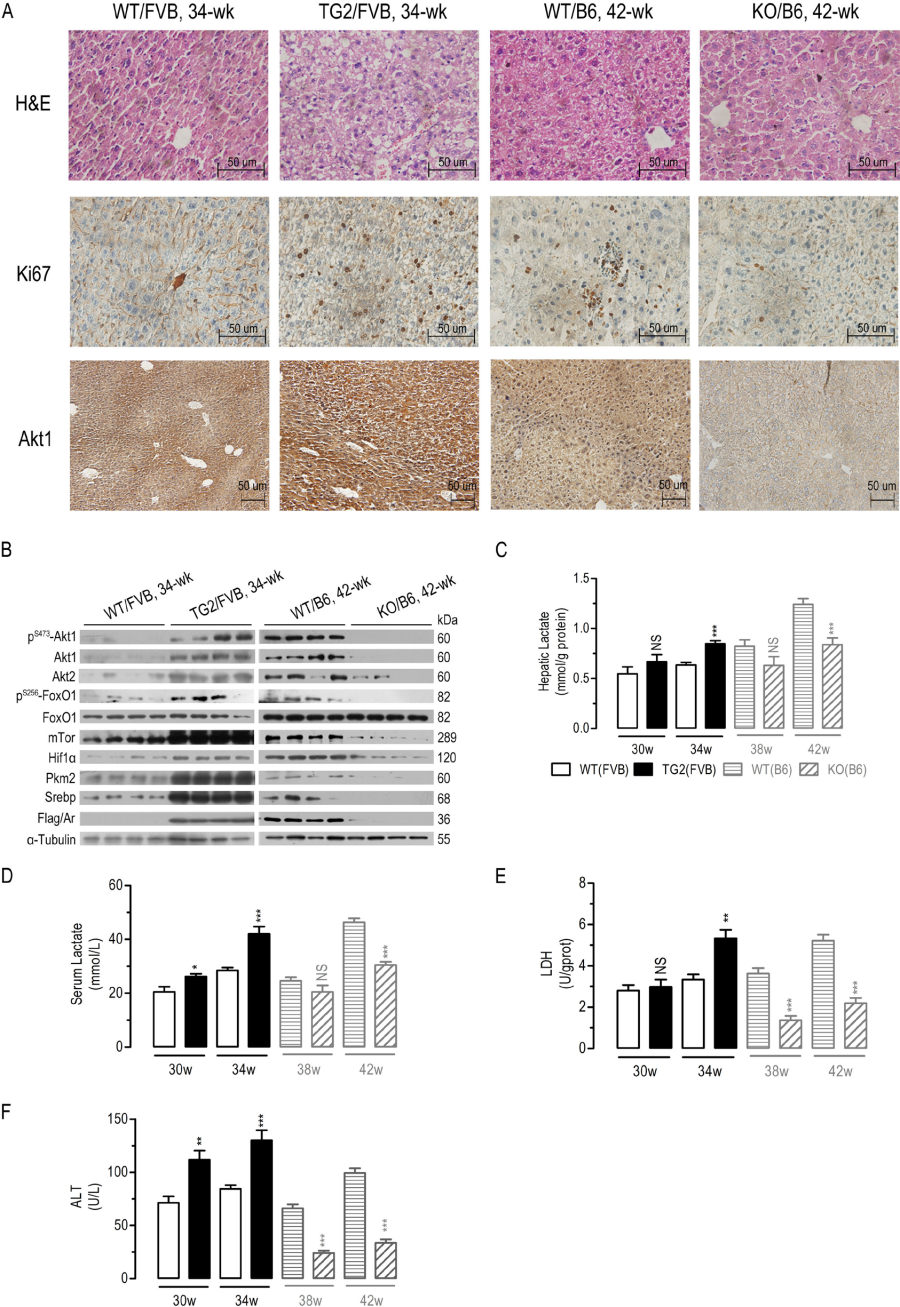
*In vivo* effects of liver-specific AR overexpression or Ar deficiency on mouse Akt/mTor signaling, LDH activity, and serum and hepatic lactate concentration (**A**) Hepatic protein expression of Ki67/Akt1 in four groups of DEN-treated mice as analyzed by immunohistochemistry (A). Hepatic protein expression of Akt/mTOR signalingin four groups of DEN-treated mice (**B**) (*n* = 4). Hepatic lactate levels (**C**) (*n* = 6–8), serum lactate levels (**D**) (*n* = 6–8), hepatic LDH activity (**E**) (*n* = 6–8), serum ALT levels (**F**) (*n* = 6–8) in eight groups of DEN-treated mice. Numeric data were expressed as the mean ± SEM. NS, not significant; *p < 0.05; **p < 0.01; ***p < 0.001, compared to WT/FVB or WT/B6 respectively.

As demonstrated by immunohistochemical (IHC) analyses, liver-specific *AR* overexpression significantly increased hepatic Akt1 expression, whereas *Ar* deficiency significantly reduced hepatic Akt1 expression in DEN-treated mice (Figure 6A). Moreover, *AR* overexpression increased cell proliferation marker Ki67, whereas Ki67 significantly suppressed in *Ar* deficient mice (Figure 6A). In *AR* overexpression mice exposed to DEN for 34 wk, hepatic protein levels of mouse p^S473^-Akt1, total Akt1/Akt2, p^S256^-FoxO1, mTor, Hif1α, Pkm2 and Srebp significantly increased (Figure 6B). Conversely, in *Ar* deficient mice exposed to DEN for 42 wk, the assayed proteins significantly reduced, except FoxO1.

Also consistent with the *in vitro* studies, *AR* overexpression increased serum and hepatic lactate concentration, liver LDH and ALT activities, and hepatic *Tnfα/Il-6* mRNA expression (Figure 6C–6F and Supplementary Figure 6), although the differences in hepatic lactate, LDH activity and *Tnf*a were not significantly of 30 wk. Conversely, *Ar* knockout decreased serum and hepatic lactate concentration, liver LDH and ALT activities, and hepatic *Tnfα/Il-6* mRNA expression (Figure 6C–6F and Supplementary Figure 6), with the exception that the differences in *Tnfα* mRNA, serum and hepatic lactate were not significantly of 38 wk. In general, these data indicated that liver AR expression regulate Akt/mTor signaling, lactate formation, and *Tnfα/Il-6* expression *in vivo*.

## DISCUSSION

Dozens of studies have reported that abnormal AKT1 activates in diabetes, cardiovascular diseases and various cancers [49–51]. Novel protein interactions with AKT/mTOR pathway members have been commonly reported to efficiently regulate AKT1 kinase activity in cancers [39, 52]. In the present study we found that overexpressed AR interacted with AKT1 to increase AKT/mTOR signaling, which in turn promoted Warburg effects, lactate production, oxidative stress, and inflammation and thus contributed to hepatocarcinogenesis (Figures 1 and 4). A series of co-immunoprecipitation assays established a protein-protein interaction between AR and the kinase domain of AKT1 (Figure 3), leading to the stabilization AKT1 (Figure 2D and 2E) and eventually significant augmentation of AKT/mTOR signaling (Figure 2A). As a consequence of its interaction with AKT1, the overexpressed AR augmented AKT/mTOR signaling (Figures 2A, 2C and 6D) and tended to enhanced lactate formation and hepatic inflammation (Figures 4 and 6). Conversely, *AR* knockdown suppressed lactate formation and inflammation. In cultured HepG2 cells, we further demonstrated that AKT1 was essential for AR-induced dysregulation of AKT/mTOR signaling, metabolic reprogramming, antioxidant defense and inflammatory responses (Figure 4). *W*e also clearly demonstrated that liver-specific *AR* overexpression leads to abnormal augmentation in hepatic AKT/mTOR signaling (Figure 6A) and enhanced HCC development (Figure 5). By contrast, oncogenic AKT/mTOR signaling and HCC development appears to be significantly ameliorated in mice deficient in *Ar*, with a few minor exceptions. Together, these data suggest that aberrantly overexpressed/over-activated hepatic AR promotes HCC development, at least in part by interacting with the oncogenic AKT1 to augment AKT/mTOR signaling.

Cancer cell metabolism is characterized by the so-called Warburg effects, the manifestations of which include enhanced glucose uptake and glycolysis, reduced oxidative phosphorylation and increased lactate secretion [53]. What might have long been overlooked, however, is the fact that the high intracellular glucose present in cancer cells very likely will trigger the overexpression of AR and/or over-activation of PP. It has been estimated that when glucose is abundant, more than 30% of glucose can be channeled into the AR/PP, which can lead to the over-production of fructose [2]. In mammalian cells, fructose also has a tendency to be converted into lipid, uric acid and lactate [54]. In this regard, it is very likely that overexpression of AR/over-activation of PP in cancer cells contribute significantly to cancer-associated metabolic reprogramming, in part by increasing synthesis of lipid, lactate and uric acid. In this investigation, we clearly demonstrated that AR overexpression promotes, and AR inhibition inhibits, lactate formation. The increase in lactate secretion from cancer cells, on the other hand, might be attributable to two distinct mechanisms: 1) high glucose directly activates AR/PP leading to the over-production of fructose and lactate; 2) overexpressed AR interacts with AKT1 to augment AKT/mTOR, HIF1a and PKM2 signaling, eventually leading to increased flux through the aerobic glycolysis to enhance lactate formation. Lactate secretion due to the over-activation of AR/PP or the augmented AKT/mTOR signaling due to AR-AKT1 interaction in cancer cells therefore might account for a significant portion of the total lactate formation, which was attributed mostly to the Warburg effects previously.

More interestingly, some reports showed that AR/AKR1B10 overexpression promotes the development of resistance against various chemotherapeutic drugs [4, 55, 56]. In addition, various studies were revealed that increased expression of AR and AKR1B10 is involved in carcinogenesis and drug resistance [10, 11, 55], and AR/AKR1B10 inhibitors could be potentially effective drugs for cancer therapeutics [55, 56]. Zopolrestat, an AR/AKR1B10 inhibitor, was found to provide additional therapeutic effects in liver cancer [57]. Epalrestat, an AKR1B1 inhibitor, significantly suppresses cancer stem cell properties, tumorigenicity, and metastasis of basal-like breast cancer cells through regulating the NF-κB pathway [58]. Consistent with previous observations, AR knockdown was found to increase susceptibility to chemotherapeutic agents, while AR expression led to tumor cell resistance to anticancer drugs (Supplementary Figure 7). Therefore, suppression of AR by inhibitors or siRNAs has the potential to serve as an adjuvant therapeutic strategy for cancers [59]. Until very recently, however, no AR inhibitor was evaluated in clinical human cancer therapy. Fidarestat, another AR inhibitor, has already been passed through the FDA's Phase-III clinical trials and has proven safe for human use, without irreversible toxicity [4]. Thus, this drug could soon be used for various cancer therapies, though clinical studies of combination therapies using known chemotherapeutic drugs with AR inhibitors/siRNAs are needed to further assess clinical toxicity and risks.

In summary, we demonstrated in this investigation that when overexpressed in liver cells, AR may mediate over-activation of PP, causing over-production of fructose, lactate, and ROS and altered expression of inflammatory response genes. Overexpressed hepatic AR may also interact with AKT1 to augment AKT-mTOR signaling, further promoting metabolic reprogramming and dysregulation of antioxidant defense and inflammatory responses. Over-activation of the polyol pathway and AR-augmented abnormal AKT-mTOR signaling may act synergistically to promote tumorigenesis in the liver. More importantly, interfering with AR/PP expression/activation may be an effective adjuvant strategy for clinical cancer therapy.

## MATERIALS AND METHODS

### Clinical mRNA expression analysis

Clinical *Akr1b1* or *Akr1b10* mRNA expression were analysed in cirrhosis and HCC on www.oncomine.org. Searching “Akr1b1” or “Akr1b10” gene expression in Mas Liver Database (GSE14323) with the following parameters “clinical specimen” in sample type, “mRNA” in Data type, “Liver cancer” in cancer type.

### Liver-specific human AR overexpressing transgenics and Ar deficient knockout mice

The liver-specific human *AR*-overexpressing transgenic FVB (TG1/FVB and TG2/FVB) mice were generated by Dan Song at Yun-qing Yang's lab of Xiamen University. The liver-specific human *AR*-overexpressing transgenic FVB (TG1/FVB and TG2/FVB) mice, *Ar* knockout C57BL/6 mice (KO/B6) and their controls (WT/FVB or WT/B6) were also kind gifts from Prof. Yun-qing Yang and only used for this project.

Mice were bred and maintained under a standard 12–12 h light-dark cycle, and were fed standard rodent chow and water ad libitum, and housed in the barrier facility of the Laboratory Animal Center, Xiamen University. All animal experiments were performed according to the protocols approved by the Institutional Animal Use and Care Committee of Xiamen University, China.

### Hepatocarcinoma (HCC) induction in mice

For the induction of HCC, the male mice of liver-specific human *AR*-overexpressing transgenic FVB (TG1/FVB and TG2/FVB), *Ar* knockout C57BL/6 (KO/B6) and their controls (WT/FVB or WT/B6) were injected intraperitoneally with diethylnitrosamine (DEN) at 25 mg/kg body weight at the age of 2 wk (Cat# 049k1613v, Sigma-Aldrich, St. Louis, MO, USA) [60]. DEN-treated TG/FVB and WT/FVB male mice were sacrificed either 30 or 34 wk of age, while DEN-treated KO/B6 and WT/B6 male mice were sacrificed either 38 or 42 wk of age. Liver lobes were photographed and tumors > 1 mm in diameter on liver surface were counted. The diameters were measured using a vernier caliper. Liver tissues were also dissected for further analyses. The phenotype data of two independent DEN-induced HCC mice models were analysed together in Figure 5. All experimental procedures involving animals were performed in accordance with the animal protocols approved by the Laboratory Animal Center of Xiamen University.

### Other procedures

All of the other procedures are established standard techniques and are described in the Supplementary Files.

### Statistical analyses

All statistical analyses were performed with the GraphPad Prism 5.0 software. Values are expressed as the means ± SEM. The Student's *t*-test (two-tailed) for pair-wise comparisons. A probability value (*p*) < 0.05 was considered to be significant, those < 0.01 or < 0.001 more so.

## SUPPLEMENTARY MATERIALS FIGURES AND TABLES



## Abbreviations

AKR1B10: aldo-keto reductase family 1B10
AKT: AR-like-1
PKB: protein kinase B
ALT: alanine aminotransferase
AMPK: AMP-activated kinase
AR: aldose reductase, aldo-keto reductase family 1B1
DEN: diethylnitrosamine
EGF: epidermal growth factor
FOXO1: forkhead box protein O1
HIF1α: hypoxia-inducible factor-1alpha
IL-6: interleukin 6
LDH: lactate dehydrogenase
mTOR: mechanistic target of rapamycin
PI3K: phosphatidylinositide 3-kinase
PKM2: pyruvate kinase isozymes M2
TNFα: tumor necrosis factor alpha
