# Supplementary material for: Aldose reductase interacts with AKT1 to augment hepatic AKT/mTOR signaling and promote hepatocarcinogenesis
Source: Oncotarget. 2017 May 10;8(40):66987–7000. doi: 10.18632/oncotarget.17791 (PMC5620151; doi:10.18632/oncotarget.17791)
Supplement: Supplementary file 1 [file oncotarget-08-66987-s001.pdf]

Aldose reductase interacts with AKT1 to augment hepatic AKT/mTOR signaling and promote hepatocarcinogenesis

SUPPLEMENTARY MATERIALS

Chemicals and plasmids

3-(4,5-dimethylthiazol-2-yl)-2,5-diphenyltetrazolium bromide (MTT, Cat# 108499), epidermal growth factor (EGF, Cat# E9644), phosphatidylinositide 3-kinase (PI3K) inhibitor (LY294002, Cat#L9908), MG132 (Cat# M7449), diethylnitrosamine (DEN, Cat# N0756), 2′,7′-dichlorodihydrofluorescein diacetate (DCFH-DA, Cat# D6883), DMSO (Cat# D2650) were purchased from Sigma-Aldrich (St. Louis, MO, USA). Protein A/G agarose (Protein A Resin Cat# L00210 and Protein G Resin Cat# L00209 were mixed 1:1) were purchased from Genscript, Nanjing, Jiangsu, China. Normal rabbit IgG (Cat# sc-2763) were purchased from Santa Cruz, CA, USA.

The anti-sense oligonucleotides (siRNAs) for human AKT1 (NM_00101443.1) were purchased from GenePharma (Shanghai, China). The targeted sequences for AKT1 siRNAs and control siRNA were 5′-AGGAAGUCAUCGUGGCCAATT-3′ (siAKT1) and 5′-UUCUCCGAACGUGUCACGUTT-3′ (siControl) respectively.

The radio-immunoprecipitation assay (RIPA) buffer for ubiquitination assay was prepared using the recipe: 0.2% SDS, 0.5% sodium deoxycolate, 0.5% Nonidet P-40, 10 mM NaF, 20 mM β-glycerophosphate, 1 mM sodium orthovanadate, 1 mM Phenylmethanesulfonyl fluoride (PMSF), 10 μg/ml leupeptin, and 2 μg/ml aprotinin.

Plasmid construction

A few pcDNA3.3-HA, pcDNA3.3-Flag, pET28a-His and pGEX-4T1-GST based vectors, an HA-tagged human AKT1 overexpressing plasmid (pHA-AKT1), a MYC-ubiquitin fusion protein overexpressing plasmid (pMYC-ubiquitin), a T308D/S473D mutant AKT1 (thus constitutively active) overexpressing plasmid (pHA-AKT1$^{T308D/S473D}$) and a T308A/S473A mutant AKT1 (thus constitutively inactive) overexpressing plasmid (pHA-AKT1$^{T308A/S473A}$), were kind gifts from Prof. Sheng-Cai Lin of Xiamen University.

Recombinant vectors expressing small hairpin RNA (shRNA) against human AR (NM_001628.2) were constructed by inserting chemically-synthesized double-strand DNA fragments containing AR-targeting shRNA sequences as listed in Supplementary Table 1 into-plasmid pLentiLox3.7 at the HapI and XhoI sites, generating plasmids pLV-shAR-1, pLV-shAR-2, pLV-shAR-3 and pLV-ctrl.[1, 2] The inserted DNA fragment was verified by DNA Sanger sequencing.

A 951-bp DNA fragment containing of human AR cDNA was isolated and amplified from human hepatocyte L-02 cells (Cat#GNHu-6, the Cell Bank of the Chinese Academy of Sciences, Shanghai, China), using primers listed in Supplementary Table 2. The PCR products were digested with HindIII-XbaI and sub-cloned into pFlag-CMV2 (Cat# E7033, Sigma-Aldrich, St. Louis, MO, USA) to generate pFlag-AR. Truncated AR cDNA fragments were PCR-amplified from pFlag-AR with primers listed in Supplementary Table 2. The PCR amplified fragments with deletions for amino acids 1–100, 101–200 and 201–316 were subcloned into pcDNA3.3-Flag by ligation independent cloning, generating pFlag-AR$^{\Delta 1–100}$, pFlag-AR$^{\Delta 101–200}$ and pFlag-AR$^{\Delta 201–316}$. Similarly, the full length AR DNA fragment was released from pFlag-AR and subcloned into pcDNA3.3-HA or pGEX-4T1-GST, generating pHA-AR and pGEX-GST-AR. The inserted DNA fragment was verified by DNA sequencing.

Truncated AKT1 cDNA fragments were PCR-amplified from the full length AKT1 (pHA-AKT1) with primers listed in Supplementary Table 2. The domain structures of human AKT1 were as shown in Supplementary Figure 3. The PCR amplified fragments containing amino acids 1-148 (Δ149–480, PH domain only), 1-412 (D413-480, PH domain + KD domain), 149-412 (Δ1-148/D413-480, KD domain only), and 149-480 (Δ1-148, KD domain + RD domain) were subcloned into pcDNA3.3-HA, generating pHA-AKT1$^{\Delta 149\text{-}480}$, pHA-AKT1$^{D413\text{-}480}$, pHA-AKT1$^{\Delta 1–148/413\text{-}480}$ and pHA-AKT1$^{\Delta 1\text{-}148}$. Similarly, the full length AKT1 DNA fragment was released from pHA-AKT1 and subcloned into pET28a-6´His or pcDNA3.3-Flag to generate pET-His-AKT1 and pFlag-AKT1. On the other hand, the Quickchange Site-Directed Mutagenesis method was used to create a KD domain deletion mutant pHA-AKT1$^{\Delta 149\text{-}412}$ using the primer sets listed on Supplementary Table 2, following the manufacturer's instructions. The mutant clones were verified by restriction enzyme digestion analyses and DNA Sanger sequencing.

Cell proliferation, migration, invasion and wound-healing assays

Cell proliferation was analyzed by the MTT (3-(4,5-dimethylthiazol-2-yl)-2,5-diphenyltetrazolium bromide) assay. Briefly, a total of 1.0´ 10^3 cells were seeded in 96-well Costar plates and 20 µl of MTT solution (5 mg/ml in PBS) was added to selected well every 24 hours for 3 days. The plates were incubated for 4 hours at 37°C in 5% CO_2 and then carefully removed medium followed by adding the solvent DMSO. The absorbance was measured at 490 nm using a microplate reader.

For colony formation assays, a total of 500 HepG2 or 1,000 SMMC7721 cells were seeded in six well plates, with medium was changed every three days. Cells were fixed with methanol and were stained with violet after 3 wk. Colony counts were counted and analyzed by Prism 5.0 software.

Cell migration and invasion assays were performed using Millicell inserts (Cat# 3422, Costar, NJ, USA) coated with matrigel (Cat# 356234, BD Biosciences, NJ, USA). HepG2 cells transfected with indicated plasmid were starved 12 h, and 1.0´ 10^6 cells were seeded per upper chambers in serum-free DMEM whereas the lower chambers were loaded with DMEM containing 10% FBS. After 48 h, the non-migrating cells on the upper chambers were removed by a cotton swab, and cells invaded through the matrigel layer to the underside of the membrane were stained and counted. Cell migration assays were performed similarly, but without matrigel gel.

For wound healing assays, HepG2 cells were transfected with plasmids as indicated. Then cells were cultured as confluent monolayers, and wounded by 20 µl pipette tip. Wounded monolayers were washed twice with PBS to remove non-adherent cells. The cells were then incubated in fresh media containing 3% fetal bovine serum (FBS) and in the presence or absence of phosphotidylinsitol-3-kinase (PI3K) inhibitor LY294002 (25 mM) or epidermal growth factor (EGF, 50 ng/ml). Cell migration was recorded under inverted microscope.

For drug resistance, a total of 4.0×10^4 cells were seeded in 96-well plates. 10 uM or 15 uM Sorafenib were added for 48 hours. MTT was added to each well and follow procedure was the same with cell proliferation assay.

Cells, cell culture and transfection

Human hepatocellular carcinoma HepG2 and human embryonic kidney HEK293T cells were purchased from ATCC (Manassas, VA, USA) and cultured as instructed. Human hepatocellular carcinoma SMMC-7721 cells were purchased from Cell Bank of the Chinese Academy of Sciences (Shanghai, China) and cultured as instructed.

Plasmid DNA transfection in mammalian cells was performed with Lipofectamine-2000 reagent (Invitrogen, Carlsbad, CA, USA) according to the manufacturer's instructions. To transiently overexpress human *AR*, HepG2 were transfected with an *AR*-overexpressing plasmid (pFlag-AR or pHA-AR) or their controls. To knock-down *AR*, HepG2 cells were transfected with plasmids carrying shRNAs for *AR* (pLV-shAR-1, pLV-shAR-2, and pLV-shAR-3) or a negative control (pLV-ctrl). To transiently overexpress *AKT1* in mammalian cells, HepG2 were transfected with an *AKT1*-overexpressing plasmid (pFlag-AKT1 or pHA-AKT1) or their controls. To knock-down *AKT1*, chemically-synthesized siRNAs or its control (GenePharma, Shanghai, China) were used to transfect HepG2 cells. Transfected cells were usually incubated for 24–72 h to allow overexpression or knock-down unless indicated otherwise.

Protein-protein interaction analyses

To analyze the interactions of plasmid-encoded AR and AKT1 in mammalian cells, HEK293T cells were co-transfected with pFlag-AR and pHA-AKT1 with Lipofectamin-2000 in 60 mm dish and incubated for 48 h. Cell lysates were immunoprecipitated with either anti-Flag or anti-HA antibody followed by Western blot analyses.

For *in vitro* AR-AKT1 binding, His-tagged *AKT1*-overexpressing pET-His-AKT1, GST-tagged *AR* overexpressing plasmid pGEX-GST-AR and its control (pGEX-4T1-GST) were used to transform *E. coli* BL21 respectively. The lysate for pET-His-AKT1-transformed cells were mixed with the lysate of either pGEX-GST-AR or pGEX-4T1-GST transformed cells. Glutathione-sepharose beads (Cat# 20211, Thermo Fisher Scientific Inc., Waltham, MA, U.S) were subsequently added to the mixtures. Bead mediated pull-down was performed as instructed and the eluates were analyzed by Western blots using anti-GST or anti-His antibody.

To map the protein domains responsible for the putative AR-AKT1 interaction, 5 deletion mutants for AKT1 and 3 deletion mutants for AR were created (Table S2). Co-immunoprecipiation was performed as described above using lysates for HEK293T cells co-overexpressing wild-type (WT) AR with AKT1 mutants or cells co-overexpressing WT AKT1 with AR mutants.

AKT1 protein uibiquitination analyses

HepG2 cells were co-transfected with pMYC-ubiquitin, pHA-AKT1 and in the presence or absence of pFlag-AR or pLV-shAR-1 as indicated and incubated for 36 h. Cells were treated with MG132 (5 µM, Cat# M7449, Sangon Biotech, Shanghai, China) for 3 h and then lysed with RIPA (0.2% SDS). After boiling the samples for 10 minutes, ubiquitinated AKT1 was immunoprecipitated with anti-HA antibody and then analyzed by Western blot using anti-MYC antibody.

Western blot analyses of protein and quantitative real-time RT-PCR (qPCR) analyses of mRNAs.

Protein concentrations for cell or tissues lysates were determined by the BCA protein assay kit (Cat# 23227, Thermo Fisher Scientific Inc., Waltham, MA, U.S) or G250 (Cat# 27816, Sigma-Aldrich, St. Louis, MO, USA). Western blots were performed using antibodies listed in Supplementary Table 4, following standard protocols.

Total RNA was isolated from HepG2 cells using the Trizol Reagent (Invitrogen) according to manufacturer's instructions. Total RNA was reverse-transcribed to cDNA using ReverTrace (Cat# RR047A, Takara, Kyoto, Japan) as instructed. SYBR green based real-time quantitative RT-PCR using a High Fidelity PrimeScript™ RT-PCR Kit (Cat# QPK-201, TOYOBO, Osaka, Japan) was performed with the primers (Supplementary Table 3). 18S rRNA served as a control.

ROS analyses

ROS was assayed by DCFH-DA (Cat# D6883, Sigma-Aldrich, St. Louis, MO, USA). DCFH-DA is a cell-permeable compound. When it enters the cell its acetate group is cleaved by cellular esterases and nonfluoresent DCFH is trapped inside. Subsequent oxidation by ROS yields the fluorescent product DCF and upon excitation at 488 nm it emits green fluorescence proportional to the intracellular level of ROS. Therefore DCFH-DA is an ROS-sensitive probe that can be used to detect oxidative activity in living cells. Following transfection, cells were washed and resuspended in PBS. DCFH-DA was then added to the resuspended cells at a final concentration of 10 μmol/L in the dark in an incubator for 30 minutes and immediately used for ROS detection by a plate reader at an excitation/emission wavelength of 485/530 nm.

Analyses of lactate concentration and lactate dehydrogenase (LDH) activity and alanine aminotransferase (ALT) activity

Serum lactate and hepatic lactate were determined using a lactate quantification kit (Cat# A019, Jiancheng Bioengineering Institute, Nanjing, China) according to the manufacturer's instructions. For liver tissues, protein concentrations were determined by G250 (Cat# 27816, Sigma-Aldrich, St. Louis, MO, USA) for liver tissue lysates.

LDH activity was assayed using a LDH quantification kit (Cat# A020, Jiancheng Bioengineering Institute, Nanjing, China) according to the manufacturer's instructions.

Serum ALT activity was determined using an ALT quantification kit (Cat# C009, Jiancheng Bioengineering Institute, Nanjing, China) according to the manufacturer's instructions.

Histological and immunohistochemical analyses

For histological and immunohistochemical assays, paraffin-embedded liver tissue samples were sectioned, deparaffinized, and rehydrated. Hematoxylin and eosin (Cat# D006, Jiancheng Bioengineering Institute, Nanjing, China) staining was performed to examine liver histological alterations. Immunohistochemical staining was performed with a MaxVision DAB kit (Cat# 0014, Maxim, Fuzhou, Fujian, China) using appropriate primary antibodies according to the manufacturer's instructions.

REFERENCES

1. Tammali R, Ramana KV, Singhal SS, Awasthi S, Srivastava SK. Aldose reductase regulates growth factor-induced cyclooxygenase-2 expression and prostaglandin E2 production in human colon cancer cells. Cancer Res. 2006; 66:9705–13.
2. D'Souza DR, Salib MM, Bennett J, Mochin-Peters M, Asrani K, Goldblum SE, Renoud KJ, Shapiro P, Passaniti A. Hyperglycemia regulates RUNX2 activation and cellular wound healing through the aldose reductase polyol pathway. J Biol Chem. 2009; 284:17947–55.
3. Toker A, Marmiroli S. Signaling specificity in the Akt pathway in biology and disease. Adv Biol Regul. 2014; 55:28–38.

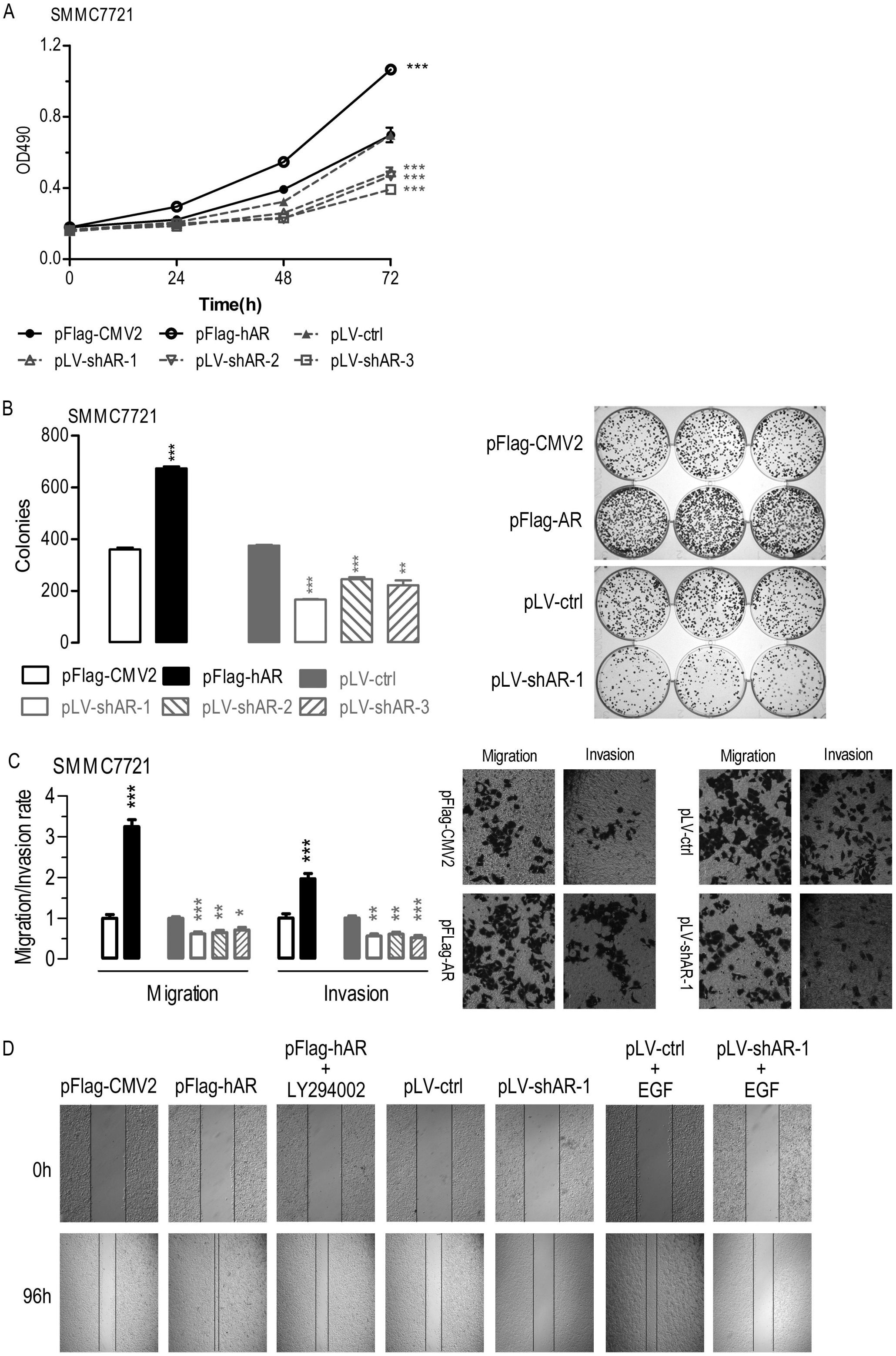

Supplementary Figure 1: Effects of AR overexpression or AR knockdown on cell proliferation, migration, invasion and colony formation in SMMC-7721 cells. Overexpression of *AR* enhanced whereas knockdown of *AR* suppressed cell proliferation (**A**) ($n = 6$), colony formation (**B**) ($n = 3$), migration and invasion (**C**) ($n = 6$), and wound healing (**D**) ($n = 3$). Data were expressed as the mean ± SEM. $^{**}p < 0.01$; $^{***}p < 0.001$, compared to pFlag-CMV2 or pLV-ctrl transfected cells.

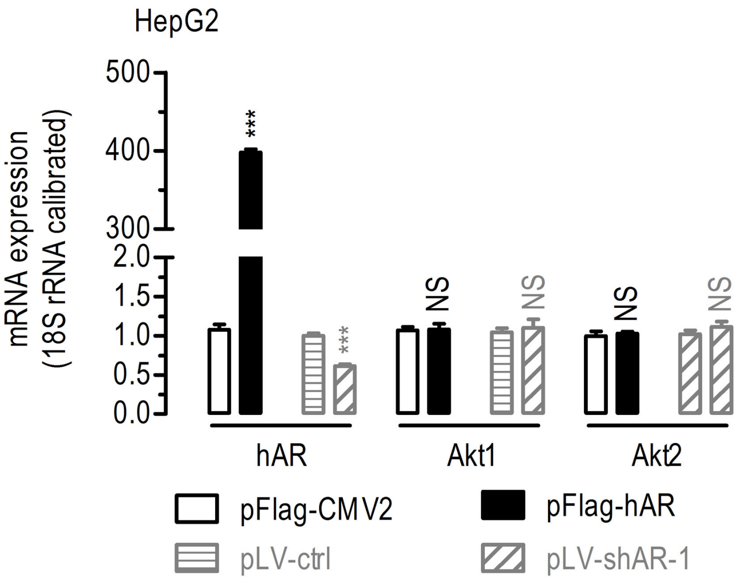

Supplementary Figure 2: AKT1/2 mRNA expression in AR-overexpressing and AR knock-down HepG2 cells. Data were expressed as the mean ± SEM, $n = 6$. NS, not significant, $***p < 0.001$, compared either to pFlag-CMV2 or pLV-ctrl transfected cells.

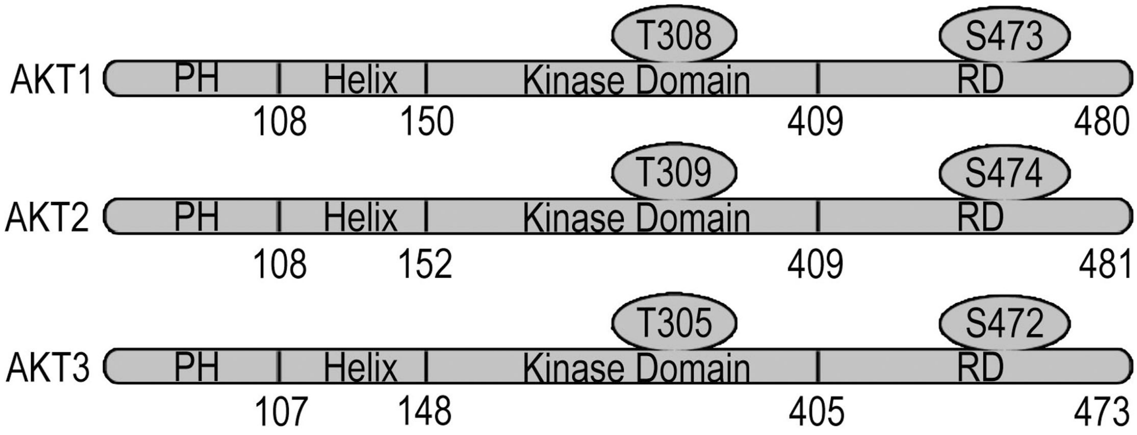

Supplementary Figure 3: A schematic graph showing the structural domains of AKT isoforms. PH, pleckstrin homology; RD, regulatory domain. Redraw after Toker and Marmiroli.[3]

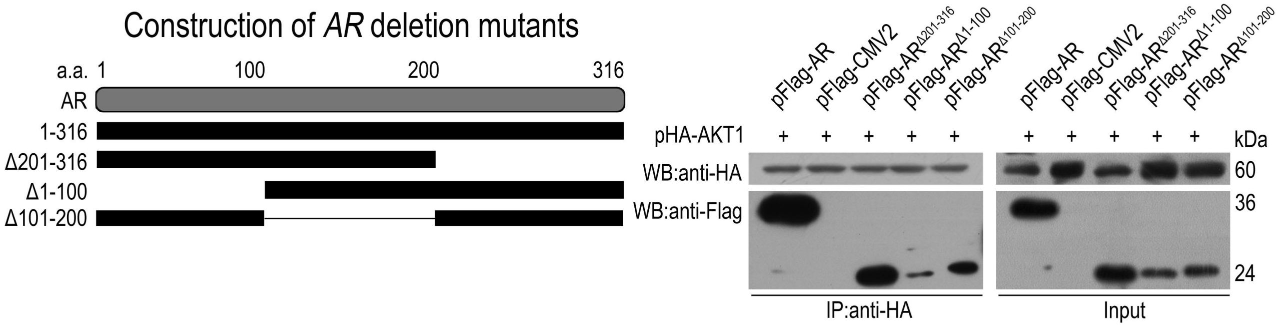

Supplementary Figure 4: Protein-protein interactions between WT AKT1 and three AR deletion mutants (Flag-AR$^{\Delta201\text{-}316}$, Flag-AR$^{\Delta1\text{-}100}$, Flag-AR$^{\Delta101\text{-}200}$).

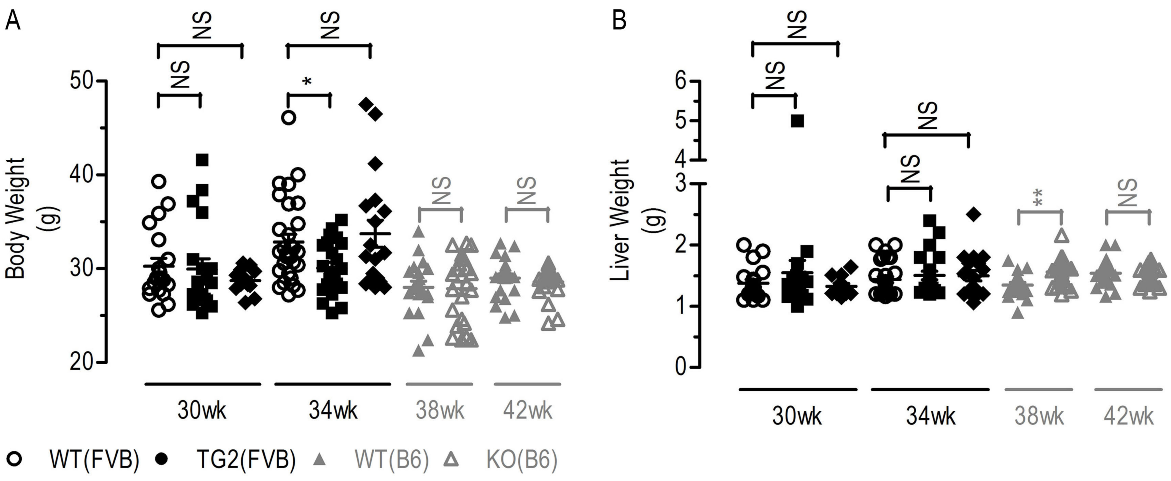

Supplementary Figure 5: *In vivo* effects of liver-specific AR overexpression or Ar deficiency on body weight, liver weight. Mice and treatments were as described in Figures 5 and 6 of the main text. Numeric data were expressed as the mean ± SEM. NS, not significant; **p* < 0.05; ***p* < 0.01; ****p* < 0.001, compared to WT/FVB or WT/B6 respectively. (**A**) Body weight in different groups of DEN-treated mice (*n* = 12–29). (**B**) Liver weight in different groups of DEN-treated mice (*n* = 12–29).

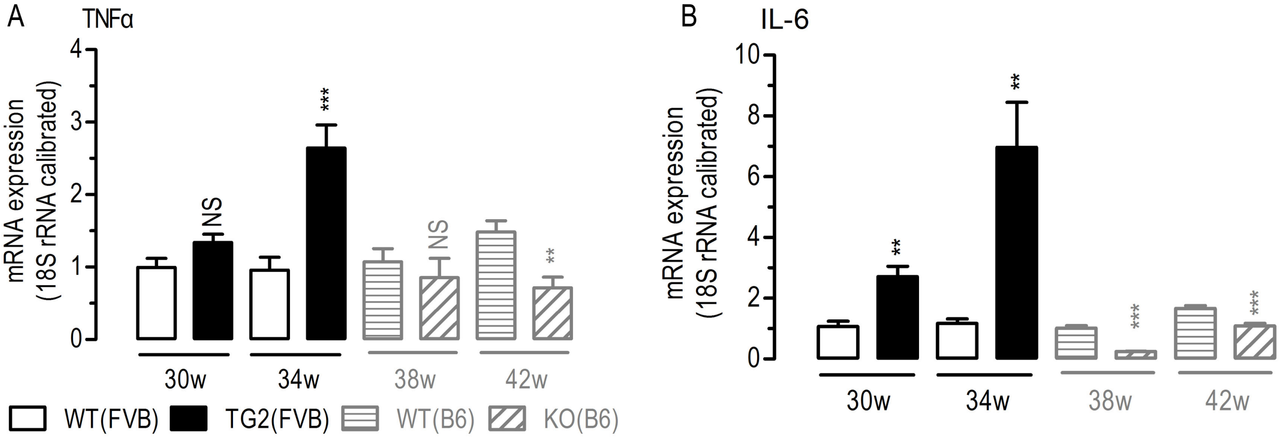

Supplementary Figure 6: *In vivo* effects of liver-specific AR overexpression or Ar deficiency on hepatic mRNA expression of Tnfa/Il-6. Mice and treatments were as described in Figures 5 and 6 of the main text. Numeric data were expressed as the mean ± SEM. NS, not significant; **p* < 0.05; ***p* < 0.01; ****p* < 0.001, compared to WT/FVB or WT/B6 respectively.

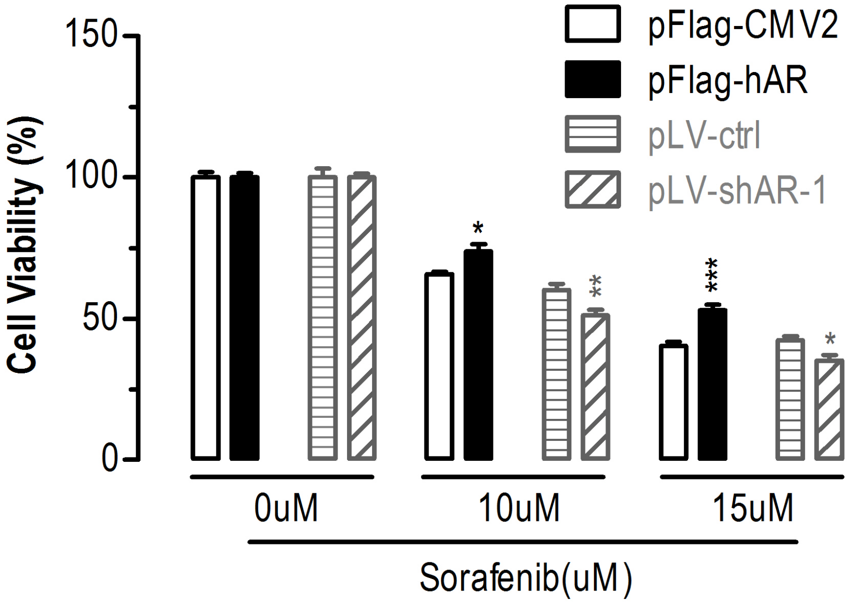

Supplementary Figure 7: AR positive-regulated drug resistance in HepG2 cells. AR overexpression increased anti-cancer drugs resistance, while AR knock-down promoted anti-cancer drugs susceptibility.

Supplementary Table 1: The targeted sequences of shRNA plasmid constructs for AR

pLV-shAR-1	5′-AATCGGTGTCTCCAACTTCAA-3′
pLV-shAR-2	5′-AACGCATTGCTGAGAACTTTA-3′
pLV-shAR-3	5′-TACCTAACTCAGGAGAAG-3′
pLV-ctrl	5′- AAAATCTCCCTAAATCATACA-3′

Supplementary Table 2: PCR or mutagenic primers used for the construction of full length or truncated mutant AR and AKT1/2 overexpressing plasmids

No.	Plasmid	Vector backbone	Primer sequence (5′→3′)	Comments
1	pHA-AKT1Δ149-480	pcDNA3.3-HA	Forward: AGAGAATTCGGATCCATGAGCGACGTGGCTATTG<br>Reverse: CTTCCATGGCTCGAGGTTCATGGTCACGCGGTG	
2	pHA-AKT1D413-480	pcDNA3.3-HA	Forward: AGAGAATTCGGATCCATGAGCGACGTGGCTATTG<br>Reverse: CTTCCATGGCTCGAGCACGATACCGGCAAAGAAG	
3	pHA-AKT1Δ1-148/413-480	pcDNA3.3-HA	Forward: AGAGAATTCGGATCCGAGTTTGAGTACCTGAAGC<br>Reverse: CTTCCATGGCTCGAGCACGATACCGGCAAAGAAG	
4	pHA-AKT1Δ1-148	pcDNA3.3-HA	Forward: AGAGAATTCGGATCCGAGTTTGAGTACCTGAAGC<br>Reverse: CTTCCATGGCTCGAGTCAGGCCGTGCCGCTGG	
5	pHA-AKT1Δ149-412	pcDNA3.3-HA	Forward: GTGACCATGAACTGGCAGCACGTGTACGAG<br>Reverse: GTTCATGGTCACGCGGTGCACGTGCTGCCA	Mutagenic primers
6	pET-His-AKT1	pET28a-6xHis	Forward: AGAGAATTCGGATCCATGAGCGACGTGGCTATTG<br>Reverse: CTTCCATGGCTCGAGTCAGGCCGTGCCGCTGG	
7	pFlag-AKT1	pcDNA3.3-Flag	Forward: AGAGAATTCGGATCCATGAGCGACGTGGCTATTG<br>Reverse: CTTCCATGGCTCGAGTCAGGCCGTGCCGCTGG	
8	pGEX-GST-AR	pGEX-4T1-GST	Forward: AGAGAATTCGGATCCATGGCAAGCCGTCTCCTGCTC<br>Reverse: CTTCCATGGCTCGAGGACGAGCAGGCAACCACAGCT	
9	pHA-AR	pcDNA3.3-HA	Forward: AGAGAATTCGGATCCATGGCAAGCCGTCTCCTGCTC<br>Reverse: CTTCCATGGCTCGAGGACGAGCAGGCAACCACAGCT	
10	pFlag-AR	pFlag-CMV2	Forward: CTGAAGCTTATGGCAAGCCGTCTCCTGCTC<br>Reverse: ACGTCTAGAGACGAGCAGGCAACCACAGCT	
11	pFlag-ARΔ1-100	pcDNA3.3-Flag	Forward: AGAGAATTCGGATCCAAGCTGGACTACCTGGACCTC<br>Reverse: CTTCCATGGCTCGAGGACGAGCAGGCAACCACAGCT	
12	pFlag-ARΔ101-200	pcDNA3.3-Flag	1-100-XhoI:<br>Forward: AGAGAATTCGGATCCATGGCAAGCCGTCTCCTGCTC<br>Reverse: TTGGACTGCAGGTCGCTGAGTGTCTTC<br>201-316-BamHI:<br>Forward: GCGACCTGCAGTCCAAAGGCATCGTGG<br>Reverse: CTTCCATGGCTCGAGGACGAGCAGGCAACCACAGCT	
13	pFlag-ARΔ201-316	pcDNA3.3-Flag	Forward: AGAGAATTCGGATCCATGGCAAGCCGTCTCCTGCTC<br>Reverse: CTTCCATGGCTCGAGGCAGTACTGGATTAACTTC	

Supplementary Table 3: List of qPCR primers

Gene	Primer sequence (5′→ 3′)	Gene ID	Amplicon (bp)
18S rRNA	Forward: GACGACCCATTCGAACGTCT<br>Reverse: CTCTCCGGAATCGAACCCTGA	NR_003286	103
AKT1 (human)	Forward: GCTGACGGCCTCAAGAAGCA<br>Reverse: ACCTTGCCGAAAGTGCCCTT	NR_00101443.1	173
AKT2 (human)	Forward: CCGCCTGTGCTTTGTGATGG<br>Reverse: TTTCCAGCTTGATGTCGCGG	NR_001243027.2	170
AR (human)	Forward: AAGTCTGTGACACCAGAACGC<br>Reverse: ACAGACCCTCCAGTTCCTGTT	NR_001628.2	111
TNFα (human)	Forward: CCCAGGCAGTCAGATCATCT<br>Reverse: TTATCTCTCAGCTCCACGCC	NM_000594.3	145
Tnfα (mouse)	Forward:CCCTCACACTCAGATCATCTTCT<br>Reverse: GCTACGACGTGGGCTACAG	NM_013693.3	61
IL-6 (human)	Forward: ACAAATTCGGTACATCCTCGAC<br>Reverse: GAATCCAGATTGGAAGCATCC	NM_000600.3	154
Il-6 (mouse)	Forward: TAGTCCTTCCTACCCCAATTTCC<br>Reverse: TTGGTCCTTAGCCACTCCTTC	NM_031168.1	76

Supplementary Table 4: List of antibodies

Antibody	Catalog#	Supplier	Cross reactivity	Dilution
Anti-α-tubulin	sc-5286	Santa Cruz, CA, USA	Human, Mouse	1:4000
Anti-β-actin	sc-47778	Santa Cruz, CA, USA	Human, Mouse	1:4000
Anti-HA (M)	sc-7392	Santa Cruz, CA, USA		1:4000
Anti-MYC	sc-40	Santa Cruz, CA, USA		1:4000
Anti-Flag	sc-807	Santa Cruz, CA, USA		1:4000
Anti-His	SAB1306082	Sigma-Aldrich, MO, USA		1:4000
Anti-HA (R)	SAB4300603	Sigma-Aldrich, MO, USA		1:4000
Anti-pS473-AKT1	AB55022	Sangon Biotech, Shanghai, China	Human, Mouse	1:4000
Anti-AKT1	AB20056	Sangon Biotech, Shanghai, China	Human, Mouse	1:4000
Anti-AKT2	AB61251	Sangon Biotech , Shanghai, China	Human, Mouse	1:4000
Anti-AR	AB60849	Sangon Biotech, Shanghai, China	Human, Mouse	1:4000
Anti-p S256-FOXO1	9461	Cell Signaling Technology, Boston, MA, USA	Human, Mouse	1:2000
Anti-FOXO1	2880	Cell Signaling Technology, Boston, MA, USA	Human, Mouse	1:4000
Anti-mTOR	2983	Cell Signaling Technology, Boston, MA, USA	Human, Mouse	1:4000
Anti-HIF1α	3716	Cell Signaling Technology, Boston, MA, USA	Human, Mouse	1:4000
Anti-PKM2	4053	Cell Signaling Technology, Boston, MA, USA	Human, Mouse	1:4000
Anti-GST	ab9085	Abcam, Cambridge, London, UK		1:4000
